# Cholesterol Crystals Activate the NLRP3 Inflammasome in Human Macrophages: A Novel Link between Cholesterol Metabolism and Inflammation

**DOI:** 10.1371/journal.pone.0011765

**Published:** 2010-07-23

**Authors:** Kristiina Rajamäki, Jani Lappalainen, Katariina Öörni, Elina Välimäki, Sampsa Matikainen, Petri T. Kovanen, Kari K. Eklund

**Affiliations:** 1 Wihuri Research Institute, Helsinki, Finland; 2 Finnish Institute of Occupational Health, Helsinki, Finland; New York University, United States of America

## Abstract

**Background:**

Chronic inflammation of the arterial wall is a key element in the pathogenesis of atherosclerosis, yet the factors that trigger and sustain the inflammation remain elusive. Inflammasomes are cytoplasmic caspase-1-activating protein complexes that promote maturation and secretion of the proinflammatory cytokines interleukin(IL)-1β and IL-18. The most intensively studied inflammasome, NLRP3 inflammasome, is activated by diverse substances, including crystalline and particulate materials. As cholesterol crystals are abundant in atherosclerotic lesions, and IL-1β has been linked to atherogenesis, we explored the possibility that cholesterol crystals promote inflammation by activating the inflammasome pathway.

**Principal Findings:**

Here we show that human macrophages avidly phagocytose cholesterol crystals and store the ingested cholesterol as cholesteryl esters. Importantly, cholesterol crystals induced dose-dependent secretion of mature IL-1β from human monocytes and macrophages. The cholesterol crystal-induced secretion of IL-1β was caspase-1-dependent, suggesting the involvement of an inflammasome-mediated pathway. Silencing of the NLRP3 receptor, the crucial component in NLRP3 inflammasome, completely abolished crystal-induced IL-1β secretion, thus identifying NLRP3 inflammasome as the cholesterol crystal-responsive element in macrophages. The crystals were shown to induce leakage of the lysosomal protease cathepsin B into the cytoplasm and inhibition of this enzyme reduced cholesterol crystal-induced IL-1β secretion, suggesting that NLRP3 inflammasome activation occurred via lysosomal destabilization.

**Conclusions:**

The cholesterol crystal-induced inflammasome activation in macrophages may represent an important link between cholesterol metabolism and inflammation in atherosclerotic lesions.

## Introduction

Chronic inflammation is recognized as a major driving force in atherogenesis [1]. The sites of atherosclerotic plaque development in the arterial wall are characterized by cholesterol accumulation and infiltration of peripheral blood monocytes, which gradually differentiate into macrophages. These cells of the innate immune system are equipped with and activated through an arsenal of receptors that detect pathogen-associated molecular patterns (PAMPs) and endogenous danger signals (DAMPs). Once activated, the monocytes and macrophages secrete a myriad of cytokines that promote inflammation in the arterial wall [2], yet the factors that trigger and maintain this cytokine release in atherogenesis are not fully understood.

Both *in vitro* and *in vivo* data show that IL-1β is a potent pro-atherogenic cytokine. In atherosclerotic coronary arteries, IL-1β levels have been shown to correlate with disease severity [3], and knocking out IL-1β in atherosclerosis-prone ApoE^−/−^ mice leads to attenuation of disease development [4]. Furthermore, IL-1β promotes the secretion of many other cytokines and chemokines and induces the expression of adhesion molecules, endothelin-1, and inducible nitric oxide synthase in endothelial cells [5]–[7].

The production of IL-1β is subject to complex regulation [8] and consequently, two separate signals are required to yield the active proinflammatory cytokine. First, induction of IL-1β mRNA, for example via stimulation of pattern recognition receptors, is needed for synthesis of proIL-1β protein in cells. A second signal is required for activation of caspase-1, a protease that cleaves proIL-1β into its biologically active secreted form [9]. Caspase-1 activation, in turn, is mediated by cytosolic protein complexes termed inflammasomes, which function in various immune cells [10]. Several different inflammasomes have been described, of which the NLRP3 (nucleotide-binding domain leucine-rich repeat containing (NLR) family, pyrin domain containing 3) inflammasome has been the most intensively studied [11]. Activated NLRP3 receptors oligomerize and recruit caspase-1 through the adaptor protein ASC (apoptosis-associated speck-like protein), thus forming the active NLRP3 inflammasome complex [11]. NLRP3 receptor is activated by diverse substances, including pore-forming toxins [12], extracellular ATP [12], viral DNA [13], inhaled particulates [14], and gout-associated uric acid crystals [15]. The highly variable structures of these substances suggest that NLRP3 receptor senses them indirectly and indeed, cellular stress signals caused by the substances have been proposed as intermediate steps in NLRP3 activation [11], [16].

Cholesterol crystals are a common, yet largely unexplored element present in atherosclerotic lesions. A sharp increase in the incidence of cholesterol crystals is observed in human atherosclerotic lesions as they progress from fatty streaks to more advanced lesions [17], [18]. It has been suggested that cholesterol crystallization may alter the mechanical properties of atherosclerotic lesions by inducing changes in lipid-pool stiffness and stability [19], [20]. Only a few studies have addressed the mechanism of cholesterol crystal formation in atherosclerotic lesions. Intracellular cholesterol crystal nucleation has been demonstrated *in vitro* in lipid-loaded macrophage foam cells [21], [22]. However, in human atherosclerotic lesions cholesterol crystals are found only rarely within cells; rather, they are typically embedded within the extracellular matrix, suggesting that nucleation of cholesterol crystals is an extracellular process [23]. As crystalline substances have been shown to induce inflammasome activation [14], [15], [24], we hypothesized that cholesterol crystals could contribute to atherogenesis through activation of the inflammasome pathway.

## Results

### Macrophages phagocytose cholesterol crystals and store crystal-derived cholesterol as cellular cholesteryl esters

**Figure 1:**
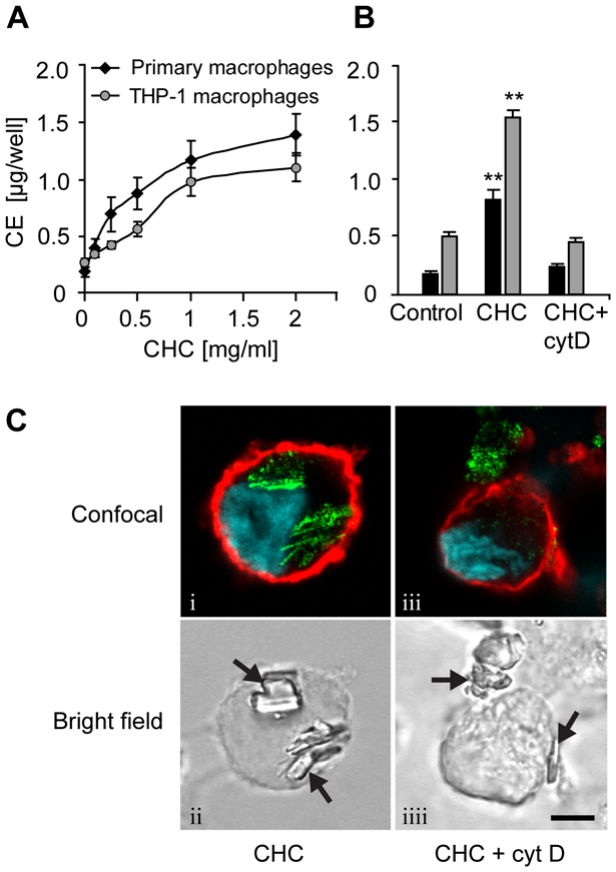
Macrophages accumulate cholesteryl esters when incubated with cholesterol crystals (CHCs). (A) Primary macrophages and THP-1 macrophages were incubated with 0.1–2 mg/ml CHCs and cholesteryl esters (CE) were measured from cellular lipid extracts by TLC. (B) Cytochalasin D (cytD; 2 µM) was employed to block cytoskeletal movements during a 16 h incubation of primary and THP-1 macrophages with CHCs (0.5 and 1.0 mg/ml, respectively). (C) THP-1 macrophages treated with CHCs ± cytD were stained with fluorophore-conjugated cholera toxin subunit B (cell membrane; red) and Hoechst (nuclei; blue). The cells were imaged using confocal fluorescence microscopy, combined with detection of CHCs by confocal reflection of the 488 nm laser line (green) (panels i, iii). CHCs are indicated by arrows in the bright field panels (ii, iiii). Scale bar 5 µm. Each experiment was performed ≥4 times. The data are means ± s.e.m. ** = p<0.01, compared to the control cells.

First, we explored whether cultured human macrophages are able to process crystalline cholesterol. For this purpose, we crystallized unesterified cholesterol from hot ethanol to obtain cholesterol crystals. Addition of the cholesterol crystals to culture medium induced a dose-dependent increase in the cholesteryl ester content of primary human monocyte-derived macrophages and PMA-differentiated THP-1 macrophages (Fig. 1A). Inhibition of cytoskeletal movements with cytochalasin D abrogated cholesterol crystal-induced cholesteryl ester accumulation (Fig. 1B), suggesting phagocytic uptake of the crystals.

To directly demonstrate that the cholesterol crystals were internalized by macrophages, confocal reflection microscopy was employed. Due to their organized molecular structure, cholesterol crystals strongly reflect the light of the excitation laser. This enables pinpointing of cholesterol crystals in thin (<0.7 µm) optical sections of cells. Representative images of THP-1 macrophages incubated with cholesterol crystals are presented in Figure 1C. Intracellular reflection signals, lacking a surrounding cell membrane, were frequently detected in THP-1 macrophages incubated with cholesterol crystals, indicating completion of phagocytosis (Fig. 1C, panels i,ii). In the presence of cytochalasin D, the cholesterol crystals remained extracellular, as evidenced by the absence of cytoplasmic reflection signals (Fig. 1C, panels iii,iiii). Together, these data demonstrate that macrophages actively phagocytosed cholesterol crystals, were able to partially dissolve them, and stored the crystal-derived unesterified cholesterol as cellular cholesteryl esters.

### Cholesterol crystals induce IL-1β secretion from human monocytes and macrophages

**Figure 2:**
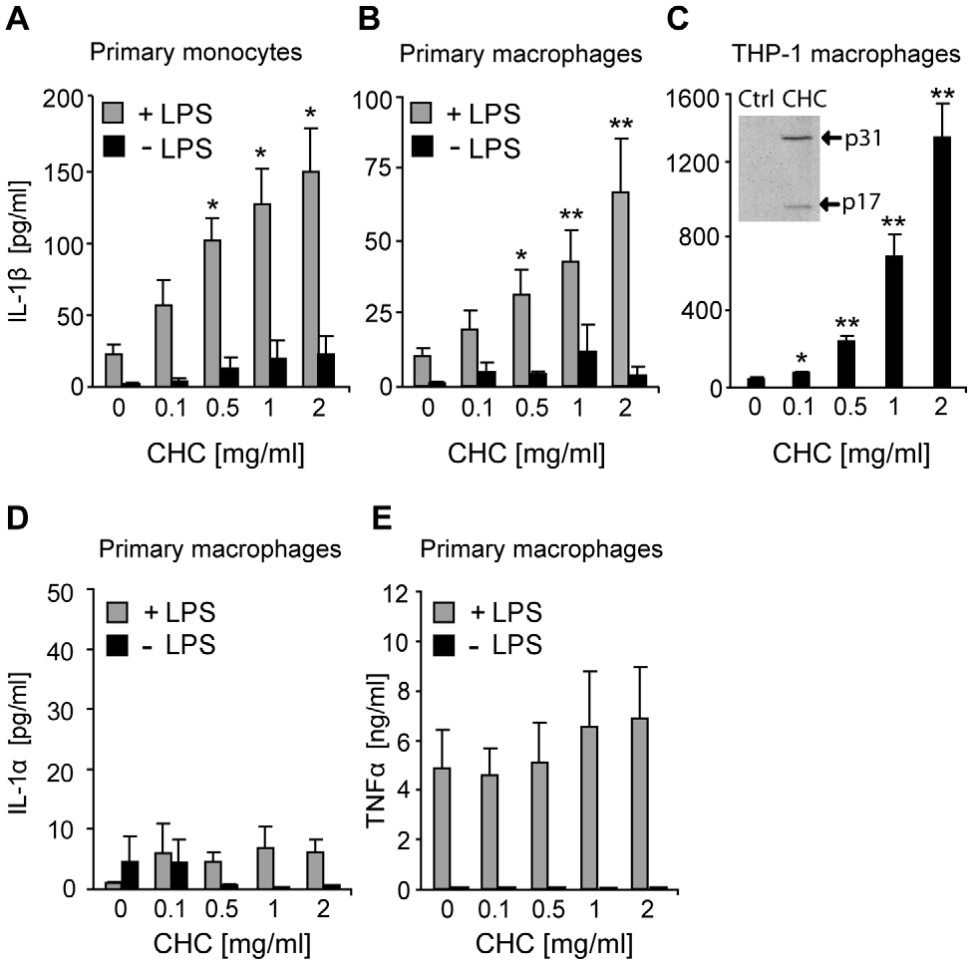
Monocytes and macrophages respond to cholesterol crystals (CHCs) by IL-1β secretion. Primary monocytes (A), primary macrophages (B,D,E), and THP-1 macrophages (C) were incubated with CHCs for 16 h, 24 h, and 8 h, respectively. LPS (1 µg/ml) was used as a co-stimulant for the primary cells. Concentrations of cytokines were subsequently determined from cell culture supernatants. The inset in 2C verifies the presence of mature 17 kDa IL-1β in cell culture supernatants of THP-1 macrophages by Western blotting. The data are means ± s.e.m. from ≥4 experiments. * = p<0.05 and ** = p<0.01, compared to untreated cells.

Next, we analyzed whether cholesterol crystals can induce cytokine secretion from monocytes and macrophages. In the presence of the Toll-like receptor (TLR) 4 ligand lipopolysaccharide (LPS), cholesterol crystals induced dose-dependent secretion of IL-1β in primary human monocytes and macrophages (Fig. 2A–B). Likewise, THP-1 macrophages exhibited a strong and rapid IL-1β response to cholesterol crystals (Fig. 2C). However, in contrast to the primary monocytes and macrophages, the IL-1β response of THP-1 macrophages was LPS-independent. In primary monocytes and macrophages, LPS induces proIL-1β mRNA and protein, whereas in THP-1 cells, PMA treatment has been shown to induce stable expression of IL-1β [25], thus eliminating the need for an additional stimulus. The presence of mature IL-1β (17 kDa) in the cell culture supernatants of cholesterol crystal-treated THP-1 macrophages was further verified by Western blot analysis (Fig. 2C, inset). Another member of the IL-1 superfamily, IL-1α, shares a common receptor and similar biological activities with IL-1β [26]–[28]. IL-1α can be secreted both as a proform and a cleaved mature form [29], but proteolysis of IL-1α is not mediated by caspase-1 [9]. We found that secretion of IL-1α by primary macrophages treated with cholesterol crystals was negligible irrespective of the presence of LPS (Fig. 2D), although LPS reportedly induces proIL-1α mRNA in macrophages [30]. Along with IL-1β, tumor necrosis factor α (TNFα) is a major pro-atherogenic inflammatory cytokine. Unlike LPS, cholesterol crystals did not induce TNFα secretion (Fig. 2E).

### Effect of cholesterol crystals and LPS on the expression of inflammasome-related genes

The known crystalline inflammasome activators induce activation of the NLRP3 inflammasome [14], [15], [24]. Moreover, upregulation of *NLRP3* receptor mRNA was recently proposed as an important checkpoint preceding NLRP3 inflammasome activation [31]. Therefore we studied whether cholesterol crystals would influence the expression of the inflammasome-related genes *NLRP3*, *NLRP1*, *CASP1* (caspase-1), and *IL1B*. Following exposure to cholesterol crystals and LPS, relative expression of the target genes in primary macrophages was analyzed by real-time quantitative RT-PCR. *TNFA* and *IL1A* were included in the analysis as positive controls for LPS-mediated induction of gene expression.

**Figure 3:**
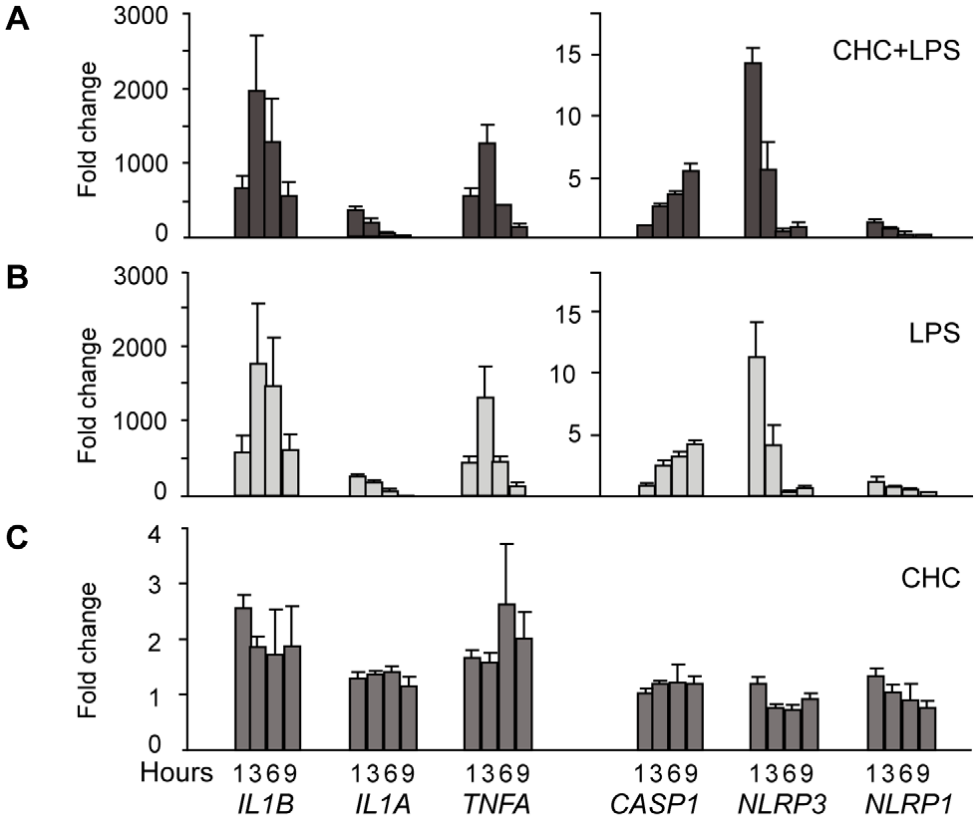
Analysis of inflammasome-related gene expression after exposure of cells to cholesterol crystals (CHCs). Primary macrophages from 3 donors were incubated for 1–9 h with (A) 0.5 mg/ml CHCs and 1 µg/ml LPS, (B) with LPS alone, or (C) with CHCs alone. Untreated control cells were included at each time point. After the incubation, mRNA levels were determined by real-time quantitative RT-PCR. The data are expressed as mean fold changes ± s.e.m. relative to the untreated cells at each time point.

*TNFA* and *IL1A* mRNAs were equally strongly induced in cells stimulated with cholesterol crystals and LPS and in cells stimulated with LPS alone, whereas cholesterol crystals alone induced only minimal changes in their expression (Fig. 3 A–C). Regarding the inflammasome-related target genes, we found that treatment with cholesterol crystals together with LPS markedly increased the expression of *NLRP3*, *CASP1*, and *IL1B* mRNAs relative to unstimulated control cells, while the levels of *NLRP1* mRNA remained low (Fig. 3A). However, a similar expression pattern was observed in cells stimulated with LPS alone (Fig. 3B) and accordingly, cholesterol crystal treatment alone induced only a minor increase in *IL1B* mRNA (Fig. 3C). Thus, the observed increases in *NLRP3*, *CASP1*, and *IL1B* expression were solely attributable to LPS, which confirms the important role of TLR signaling in setting the stage for inflammasome activation.

### Cathepsin B and potassium efflux are essential for cholesterol crystal-induced inflammasome activation

**Figure 4:**
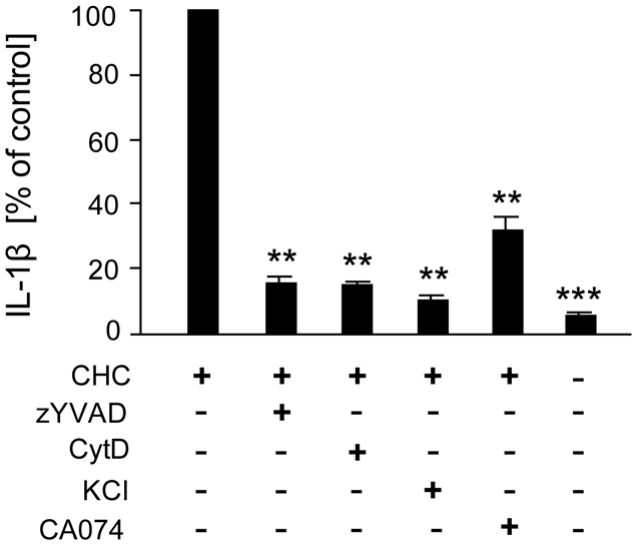
Mechanism of cholesterol crystal (CHC)-induced IL-1β secretion. THP-1 macrophages were incubated with CHCs in the absence or presence of caspase-1 inhibitor zYVAD-fmk (25 µM), cytochalasin D (2 µM), KCl (130 mM), or cathepsin B inhibitor CA-074Me (10 µM). After the incubation, cell culture supernatants were analyzed for IL-1β (average response to CHCs 662 pg/ml). The data are means ± s.e.m. from ≥5 experiments. ** = p<0.01 and *** = p<0.001, compared with CHC-treated cells.

Next, we studied the potential mechanisms of cholesterol crystal-induced IL-1β secretion in THP-1 macrophages. Firstly, caspase-1 inhibitor zYVAD-fmk abrogated cholesterol crystal-induced IL-1β secretion, confirming that IL-1β maturation was indeed inflammasome-driven (Fig. 4). Furthermore, the IL-1β response was diminished by cytochalasin D, indicating that phagocytosis of cholesterol crystals was required for inflammasome activation (Fig. 4). Potassium efflux has been linked to activation of both NLRP3 and NLRP1 inflammasomes [32]. IL-1β response to the crystals was eliminated at high extracellular potassium concentrations (Fig. 4), indicating the involvement of potassium efflux in cholesterol crystal-mediated inflammasome activation. Moreover, leakage of the lysosomal cysteine protease cathepsin B into the cytoplasm has been associated with activation of the NLRP3 inflammasome [24], although the exact mechanism of activation remains unknown. Cathepsin B inhibitor CA-074Me [33]–[35] significantly reduced the IL-1β response (Fig. 4), suggesting a role for cathepsin B and NLRP3 in cholesterol crystal-induced inflammasome activation.

### Cholesterol crystals promote destabilization of lysosomes and cathepsin B leakage into the cytoplasm

**Figure 5:**
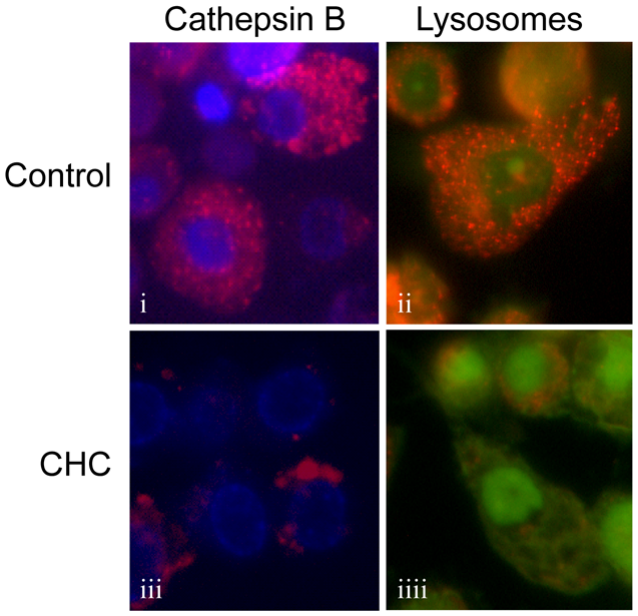
Cholesterol crystals (CHCs) cause destabilization of lysosomes and leakage of cathepsin B into the cytoplasm. CHC-treated or untreated live THP-1 macrophages were stained with cathepsin B substrate z-Arg-Arg-cresyl violet (panels i, iii) or with acridine orange (panels ii, iiii). The fluorescent cresyl violet group of z-Arg-Arg-cresyl violet is dequenched upon cleavage of one or both of the arginines by cathepsin B. Acridine orange aggregates in the acidic pH of lysosomes, which changes the fluorescence emission of the dye from green to red. The images are representative of 3 experiments.

The involvement of cathepsin B in cholesterol crystal-induced inflammasome activation prompted us to study the effect of cholesterol crystals on lysosomal integrity and cathepsin B localization. To visualize cathepsin B activity in live THP-1 macrophages, we utilized a cell-permeable fluorescently labeled cathepsin B substrate z-Arg-Arg-cresyl violet [36], [37]. Acridine orange staining was used to monitor lysosomal integrity. We found that in unstimulated cells, cathepsin B staining was bright and corresponded well to the punctuate lysosomal staining pattern seen in acridine orange-stained cells (Fig. 5, panels i,ii). In cholesterol crystal-treated cells, cathepsin B activity was markedly reduced (Fig. 5, panel iii), suggesting leakage of cathepsin B into the cytoplasm, where the enzyme is gradually inactivated by neutral pH. Supporting this idea, the red acidity-dependent acridine orange signal in lysosomes was diminished by crystal treatment, suggesting destabilization of the lysosomal membranes (Fig. 5, panel iiii).

### Cholesterol crystals activate the NLRP3 inflammasome

**Figure 6:**
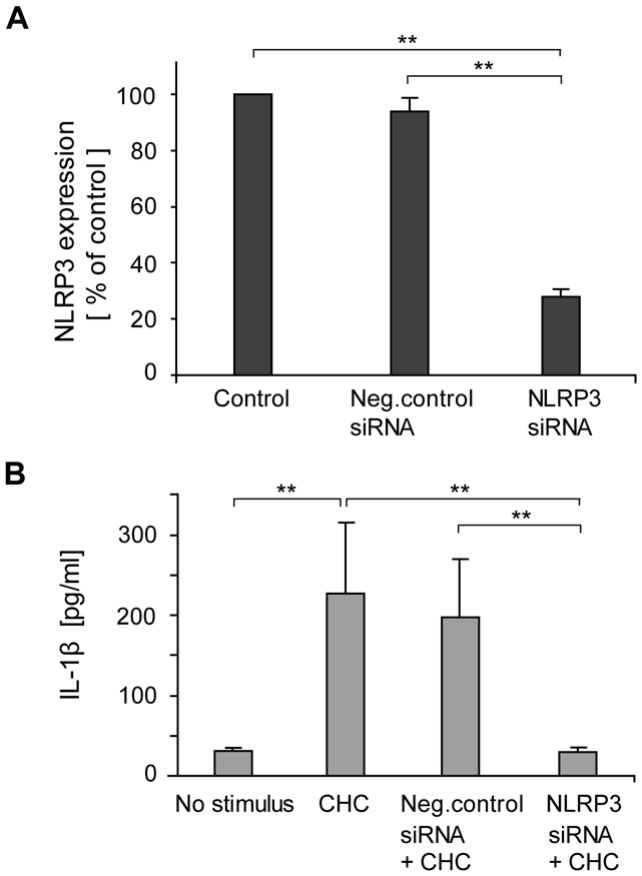
Silencing of NLRP3 attenuates cholesterol crystal (CHC)-induced IL-1β secretion. (A) NLRP3 mRNA levels were reduced by 72% after treatment of THP-1 macrophages with NLRP3-targeted small interfering RNA (siRNA). (B) CHC-induced IL-1β secretion was abolished after treatment of cells with NLRP3 siRNA, whereas treatment of cells with negative control siRNA had no effect. The data are means ± s.e.m. from 5 (A) and 3 (B) experiments. ** = p<0.01.

Collectively, the data implied involvement of the NLRP3 inflammasome in cholesterol crystal-induced IL-1β response. To verify this, expression of NLRP3 receptor, the crucial component in NLRP3 inflammasome, was silenced in THP-1 macrophages using a combination of two small interfering RNAs (siRNAs). The siRNA treatment reduced *NLRP3* expression by 72%, while negative control siRNA had no effect (Fig. 6A). Importantly, silencing of *NLRP3* gene completely abolished cholesterol crystal-induced IL-1β secretion, while no significant inhibition of IL-1β secretion was observed in cells transfected with negative control siRNA (Fig. 6B). This effect of the NLRP3 siRNA was not due to reduction in cholesterol crystal-induced lysosomal destabilization, as cholesterol crystal-induced cathepsin B leakage to cytoplasm was equally extensive in macrophages transfected with NLRP3 siRNA compared to untransfected macrophages (Fig. S1). These data confirm the role of NLRP3 receptor as the cholesterol crystal-responsive mediator of inflammasome assembly in human macrophages.

## Discussion

Cholesterol crystals formed in atherosclerotic lesions have been traditionally considered an inert material and solely an epiphenomenon of the disease. However, the present data argue that cholesterol crystals may have a significant proinflammatory role in atherogenesis. We show here that cholesterol crystals induce inflammasome activation and secretion of the highly proinflammatory cytokine IL-1β in human macrophages. This novel mode of macrophage activation in atherosclerotic lesions may represent a significant source of inflammation in the affected tissue.

Earlier studies on macrophage-cholesterol crystal interaction have suggested sequestration of cholesterol crystals in surface-connected compartments [38], as well as complete phagocytosis of the crystals [39]. We showed by confocal reflection microscopy that human macrophages phagocytose cholesterol crystals and, despite their relatively large size, are able to fully internalize the crystals. Moreover, phagocytosis of cholesterol crystals led to accumulation of cellular cholesteryl esters in macrophages. These observations indicate that macrophages possess the capacity to remove, at least to some extent, the cholesterol crystals deposited in atherosclerotic lesions. Importantly, we showed that phagocytosed cholesterol crystals can elicit an inflammatory reaction by triggering macrophage IL-1β secretion. The IL-1β response to cholesterol crystals was caspase-1-dependent, indicating inflammasome-mediated molecular mechanisms. Indeed, siRNA silencing experiments confirmed NLRP3 receptor, the initiator of NLRP3 inflammasome assembly, as the cholesterol crystal-responsive element in macrophages. These observations imply that cholesterol crystals formed in atherosclerotic lesions may not be as inert as previously thought, as they are capable of eliciting a strong inflammatory reaction.

The NLRP3 receptor can be activated by diverse agents, such as extracellular ATP [12], inhaled silica and asbestos [14], [24], and endogenously formed monosodium urate crystals [15]. Potassium efflux [32], leakage of lysosomal cathepsin B into the cytoplasm [24], and/or reactive oxygen species formation [14] caused by these substances seem to be essential intermediate steps in activation of NLRP3 receptor, although the interplay between these processes is poorly understood [11], [16]. Our data suggest that both potassium efflux and cathepsin B activity contribute to cholesterol crystal-mediated activation of the NLRP3 inflammasome. Further studies are required to elucidate the possible interconnection and relative importance of these cellular factors in cholesterol crystal-induced NLRP3 activation. It is important to note, however, that some uncertainty exists regarding selectivity of the cathepsin B inhibitor CA-074Me used in these experiments [33]–[35]. It has been shown that in murine fibroblasts CA-074Me inhibits the activity of both cathepsin B and the very closely related cathepsin L [40]. We thus conclude that both cathepsins B and L may be involved in triggering NLRP3 activation in response to cholesterol crystals. Of interest, protein levels of both cathepsin B and L are elevated in atherosclerotic plaques compared to normal arterial tissue, and colocalization with macrophage markers is observed for both cathepsins [41], [42].

Because of its proIL-1β-inducing effect, LPS-priming of primary macrophages was essential for cholesterol crystal-induced IL-1β secretion. Several lines of evidence indicate that TLR signaling is operative in atherosclerotic arterial intima, and may contribute to disease pathogenesis [43], [44]. In addition to TLRs, an abundance of TLR ligands, both bacterial [45] and endogenous [46]–[49], has been demonstrated in atherosclerotic lesions. Considering these observations, it is conceivable that cholesterol crystals, in concert with proatherogenic TLR ligands, can elicit inflammasome activation in the lesions. This is further supported by the finding that caspase-1 mRNA and protein can be found in human atherosclerotic lesions, while they are virtually absent in normal arteries [50].

**Figure 7:**
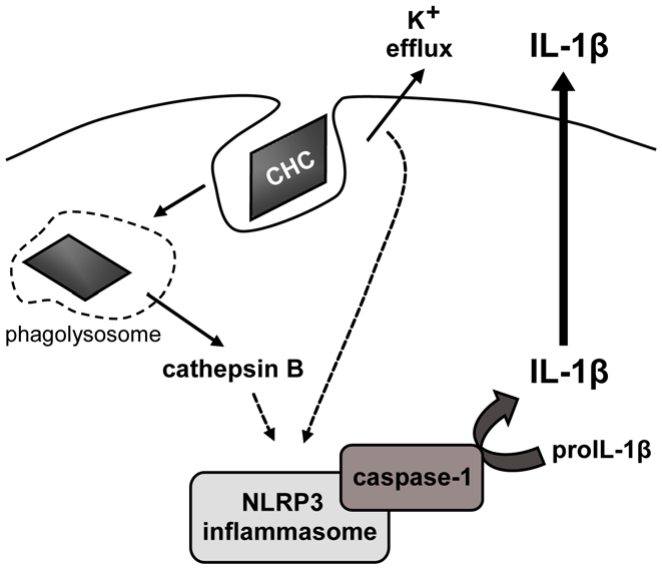
Proposed mechanism of cholesterol crystal (CHC)-induced inflammasome activation. CHCs are phagocytosed by macrophages, causing lysosomal destabilization and leakage of cathepsin B to cytoplasm, where the enzyme indirectly activates the NLRP3 inflammasome. Lowering of intracellular potassium concentration, stemming from potassium efflux caused by phagocytosed CHCs, is also required for NLRP3 activation.

Taken together, the present findings demonstrate that cholesterol crystals, common constituents of atherosclerotic lesions, induce NLRP3 inflammasome activation and IL-1β secretion in human macrophages. The suggested mechanism of NLRP3 activation by cholesterol crystals involves both potassium efflux and cathepsin B leakage into the cytoplasm (Fig. 7). In atherosclerosis, the inflammasome-mediated IL-1β release would promote an inflammatory milieu and thus drive lesion progression. Consequently, the cholesterol crystal-induced inflammasome activation may represent an important link between cholesterol metabolism and inflammation in atherosclerotic lesions.

While this study was being finalized, Duewell and colleagues published a similar study reporting that cholesterol crystals activate the NLRP3 inflammasome in mouse macrophages through a lysosomal damage- and cathepsin-mediated mechanism [51]. Our study shows that the same activation pathway of NLRP3 is functional also in human macrophages exposed to cholesterol crystals. Furthermore, Duewell and colleagues showed that cholesterol crystals can be detected already at an early stage of disease development in the atherosclerotic ApoE^−/−^ mouse model. Altogether, the results by Duewell et al. and the results of the present study strongly implicate cholesterol crystals as a potential source of inflammation in atherosclerotic lesions.

## Materials and Methods

### Ethics statement

Buffy coats were obtained from healthy human donors after informed consent as by-products from the preparation of blood products for clinical use (Red Cross Finland Blood Service, Helsinki, Finland).

### Cell culture

Buffy coats were obtained from healthy human donors (Red Cross Finland Blood Service, Helsinki, Finland). Monocytes were isolated and differentiated into macrophages as described [52], except that recombinant human macrophage colony-stimulating factor (M-CSF, 50 ng/ml; Nordic Biosite, Täby, Sweden) was used instead of GM-CSF. Human monocytic leukaemia cell line THP-1 was obtained from the American Type Culture Collection (Manassas, VA; cat. TIB-202) and maintained in RPMI 1640 supplemented with 2 mM L-glutamine, 10% fetal bovine serum, 25 mM HEPES, 100 U/ml penicillin, and 100 µg/ml streptomycin (standard THP-1 medium). To induce monocyte-to-macrophage differentiation, the THP-1 cells were cultured for 72 h in the standard culture medium supplemented with 100 nM phorbol 12-myristate 13-acetate (PMA; Sigma-Aldrich, St. Louis, MO).

### Preparation of cholesterol crystals

Cholesterol (Sigma, Saint Louis, MO; cat. C8667) dissolved in 95% ethanol (12.5 g/l) was heated to 60°C, filtered through Whatman filter paper while still warm, and left at room temperature to allow crystallization to proceed [53]. Flat, rhomboid, and relatively large (1–5 mm) cholesterol crystals were formed. The crystals were collected by filtering, autoclaved, ground using a sterile mortar and a pestle to yield a size range of 1–10 µm, and stored at −20°C until use. Endotoxin was not detected in the crystals by Limulus Amebocyte Lysate QCL-1000 assay (Lonza/Cambrex, Walkersville, MD).

### Treatment of cells with cholesterol crystals

Primary monocytes and macrophages (1.5×10^6^ mononuclear cells/well, of which ∼20% are monocytes) and THP-1 macrophages (3×10^5^ cells/well) were cultured on 24-well plates and subjected to cholesterol crystals for 4–24 h at +37°C under 5% CO_2_ in a serum-free culture medium. Where indicated, also 1 µg/ml LPS from E.coli (Sigma; serotype 0111:B4), 25 µM caspase-1 inhibitor z-YVAD-fmk (Santa Cruz Biotechnology, Santa Cruz, CA), 2 µM cytochalasin D (Sigma), 10 µM cathepsin B inhibitor CA-074Me (Calbiochem), or 130 mM KCl was added to the incubation medium. After the incubations, culture media were collected for analysis of their cytokine contents and the cells were washed with PBS and subjected to lipid extraction or RNA isolation as described below.

### Lipid extraction and analysis by thin layer chromatography

Cellular lipids were extracted from the cells with hexane-isopropanol (3∶2, v/v). The solvent was evaporated and the lipids were redissolved in chloroform-methanol (2∶1, v/v). The samples were then applied onto silica-coated thin layer chromatography (TLC) plates (CAMAG, Berlin, Germany) using an automatic TLC sampler (Sampler3; CAMAG). Hexane/diethyl ether/concentrated acetic acid/H_2_O (130∶30∶2∶0.5, v/v) was used as the mobile phase for TLC analysis of cholesterol ester content in the samples. The lipids were visualized by dipping the TLC plate into CuSO_4_ (3%)/H_2_PO_4_ (8%) and by subsequently heating the plate for 10 min at 150°C. The bands were scanned with TLC Scanner3 (CAMAG) and analyzed using TLC Evaluation Software (CAMAG).

### Analysis of cytokine secretion

IL-1β, IL-1α, and TNFα were analyzed from the culture media samples using commercial enzyme-linked immunosorbent assays (ELISA) according to the manufacturer's protocols (all ELISAs were from R&D Systems, Minneapolis, MN).

### Western blotting

Culture medium supernatants from cholesterol crystal-treated (1 mg/ml, 24 h) THP-1 macrophages were concentrated and purified using Amicon Ultra-15 centrifugal filter devices (10 kDa cut-off; Millipore, Bedford, MA) and 2-D Clean-Up Kit (GE Healthcare Life Sciences, Uppsala, Sweden). A culture medium sample corresponding to 1.5×10^6^ cells was run in polyacrylamide gel electrophoresis and transferred to an Immobilon-P Transfer Membrane (Millipore). The polyclonal anti-IL-1β antibody used for detection has been described previously [54].

### NLRP3 knock-down with small interfering RNA (siRNA)

THP-1 cells were seeded on 12-well plates (1.5×10^5^ cells/well) in the standard THP-1 culture medium supplemented with 50 nM PMA. After 24 hours, the cells were transfected with 100 nM total siRNA targeted at NLRP3 (also known as CIAS1) mRNA (Hs_CIAS1_6 and Hs_CIAS1_9 siRNAs at 1∶1 molar ratio; Qiagen, Valencia, CA) or with 100 nM AllStars Negative Control siRNA (Qiagen), using the HiPerFect transfection reagent (Qiagen) according to the manufacturer's instructions. After 22 h of incubation with the siRNAs, the cells were washed with PBS and incubated for a further 12 h in serum-free THP-1 medium without or with 1 mg/ml cholesterol crystals. Finally, the media were collected and RNA was isolated from the cells for quantitative real-time PCR.

### Quantitative real-time RT-PCR

Total cellular RNA was purified from the cells using RNeasy columns (Qiagen). RNA from each sample (0.25–0.5 µg) was converted to cDNA using MMLV reverse transcriptase and random hexamers (Promega, Madison, WI, USA). The cDNA was amplified in duplicate using TaqMan Universal PCR Master Mix (Applied Biosystems, Foster City, CA) with gene-specific primers and probes (Table 1) on ABI PRISM 7500 sequence detector system (Applied Biosystems, Foster City, CA). The data were developed with Sequence Detector System software (version 1.4, Applied Biosystems) and the threshold value (Ct) of a sample was selected according to the manufacturer's guidelines. For data normalization, an endogenous control (18S rRNA) was determined for controlling the cDNA input and the relative units were calculated by a comparative Ct method [55].

**Table 1:** Primers and probes used in quantitative real-time RT-PCR.

Primer or fluorogenic probe	Sequence
IL1B-F*	5′-TTACAGTGGCAATGAGGATGAC -3′
IL1B-R#	5′- GTCGGAGATTCGTAGCTGGAT -3′
IL1B probe	5′-FAM-AACAGATGAAGTGCTCCTTCCAGGACC-BHQ1-3′
IL1A-F	5′-ATCAGTACCTCACGGCTGCTG-3′
IL1A-R	5′-TGGGCAGTCACATACAATTGAGT-3′
IL1A probe	5′-FAM-CCCATGTCAAATTTCACTGCTTCATCCA-BHQ1-3′
TNFA-F	5′- GCTGCACTTTGGAGTGATCG-3′
TNFA-R	5′- GTTTGCTACAACATGGGCTACAG-3′
TNFA probe	5′-FAM- CCCAGGCAGTCAGATCATCTTCTCGA-BHQ1-3′
CASP1-F	5′- CCGAAGGTGATCATCATCCA-3′
CASP1-R	5′- ATAGCATCATCCTCAAACTCTTCTG-3′
CASP1 probe	5′-FAM-CCTGCCGTGGTGACAGCCCTG-BHQ1-3′
NLRP1-F	5′-GGAGGCCTTGGTGAAACC-3′
NLRP1-R	5′-CGATGTCACTCGGGCTATCA-3′
NLRP1 probe	5′-FAM- CAGCCCGCATAGCCGTACCTTCA-BHQ1-3′
NLRP3-F	5′-GGAGAGACCTTTATGAGAAAGCAA-3′
NLRP3-R	5′-GCTGTCTTCCTGGCATATCACA-3′
NLRP3 probe	5′-FAM-ACGTGCATTATCTGAACCCCACTTCGG-BHQ1-3′
18S-F	5′-CGGCTACCACATCCAAGGAA-3′
18S-R	5′-GCTGGAATTACCGCGGCT-3′
18S probe	5′-FAM-TGCTGGCACCAGACTTGCCCTC-BHQ1-3′

*forward primer.

# reverse primer.

### Fluorescence imaging of cathepsin B and lysosomes

Cholesterol crystal-treated THP-1 macrophages were stained with cell-permeable fluorescently labeled cathepsin B substrate z-Arg-Arg-cresyl violet and Hoechst stain or with 5 µM acridine orange (AO) according to the manufacturer's instructions (CV-Cathepsin B Detection Kit by BIOMOL, Plymouth Meeting, PA). The coverslips containing the stained live cells were then mounted in a drop of PBS and examined within 30 min using an epifluorescence microscope.

### Confocal reflection microscopy

THP-1 macrophages were treated with cholesterol crystals in the absence or presence of cytochalasin D. The nuclei were then stained with Hoechst, followed by staining of the cell membrane ganglioside GM1 with 12 µg/ml cholera toxin subunit B Alexa Fluor647 conjugate (CTXb-647; Molecular Probes, Eugene, OR) for 30 min at +4°C [56], [57]. Finally, the cells were fixed and the coverslips were mounted on glass slides. Images were acquired using LSM 510 Meta confocal laser scanning microscope (Carl Zeiss AG, Göttingen, Germany) with a 63× Plan-Apochromat oil immersion objective (NA = 1.4). Fluorescence signals from Hoechst and CTXb-647 were captured simultaneously with the reflection signal (excitation 488 nm, detector channel set to 475–525 nm) from cholesterol crystals.

### Statistics

Calculations were performed with PASW Statistics 17.0 (SPSS Inc., Chicago, IL) using the non-parametric Mann-Whitney's U-test. Data are presented as means ± standard error of the mean (s.e.m.). Statistical significance was set to p<0.05.

## Supporting Information

Figure S1 NLRP3 siRNA treatment does not reduce cholesterol crystal (CHC)-induced lysosomal destabilization. THP-1 macrophages were transfected with NLRP3 siRNA or left untreated. The cells were subsequently exposed to 1 mg/ml of cholesterol crystals for 6 h and stained with the fluorescently labeled cathepsin B substrate z-Arg-Arg-cresyl violet (red) and nuclear Hoechst stain (blue). For detailed protocols, refer to the materials and methods section. In control cells that received neither siRNA nor CHCs (A) cathepsin B activity localized to abundant small cytoplasmic vesicles in agreement with a lysosomal localization. In cells treated with CHCs alone (B) or with NLRP3 siRNA and CHCs (C) cathepsin B activity was markedly reduced indicating leakage of cathepsin B to cytoplasm where the enzyme is gradually inactivated by neutral pH.
(5.11 MB TIF)
