# Supplementary figures and images for: Cholesterol Crystals Activate the NLRP3 Inflammasome in Human Macrophages: A Novel Link between Cholesterol Metabolism and Inflammation
Source: PLoS One. 2010 Jul 23;5(7):e11765. doi: 10.1371/journal.pone.0011765 (PMC2909263; doi:10.1371/journal.pone.0011765)

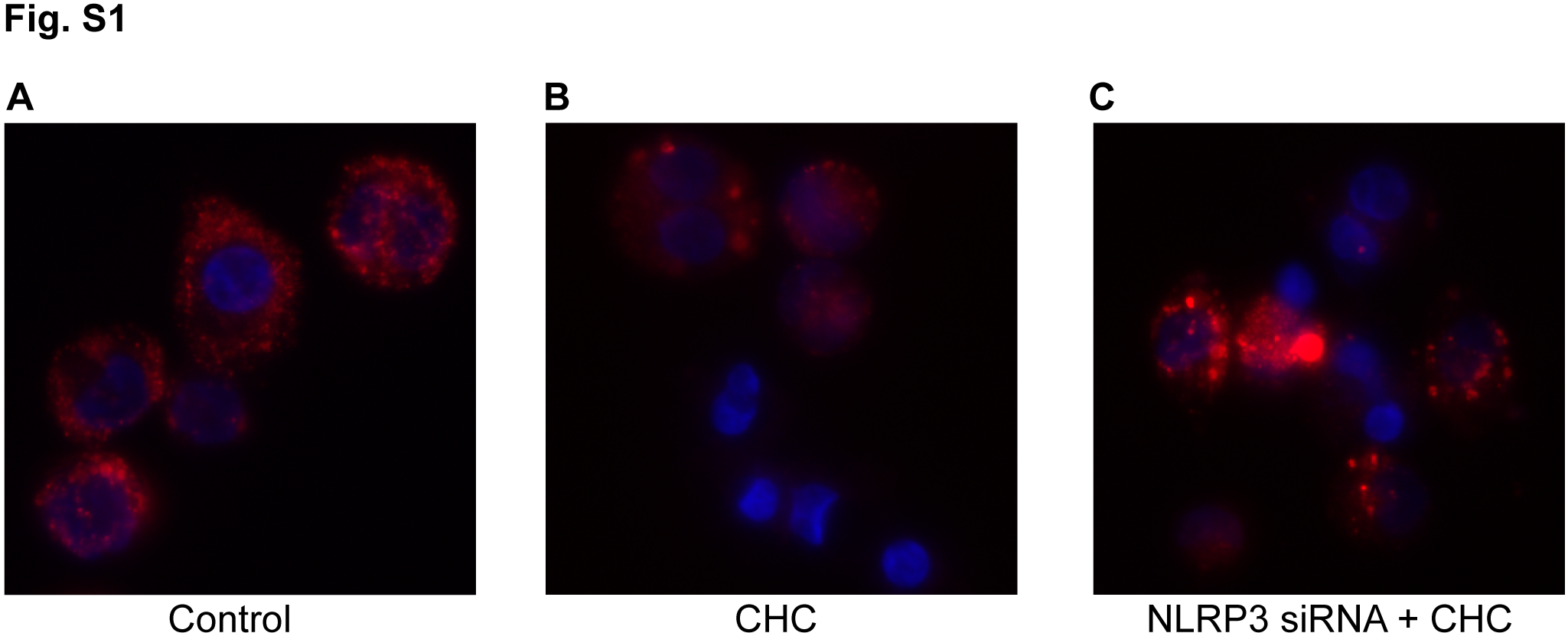

Supplement: Figure S1 — NLRP3 siRNA treatment does not reduce cholesterol crystal (CHC)-induced lysosomal destabilization. THP-1 macrophages were transfected with NLRP3 siRNA or left untreated. The cells were subsequently exposed to 1 mg/ml of cholesterol crystals for 6 h and stained with the fluorescently labeled cathepsin B substrate z-Arg-Arg-cresyl violet (red) and nuclear Hoechst stain (blue). For detailed protocols, refer to the materials and methods section. In control cells that received neither siRNA nor CHCs (A) cathepsin B activity localized to abundant small cytoplasmic vesicles in agreement with a lysosomal localization. In cells treated with CHCs alone (B) or with NLRP3 siRNA and CHCs (C) cathepsin B activity was markedly reduced indicating leakage of cathepsin B to cytoplasm where the enzyme is gradually inactivated by neutral pH. (5.11 MB TIF) [file pone.0011765.s001.tif]
